# Evaluation of serum exosomal LncRNA‐based biomarker panel for diagnosis and recurrence prediction of bladder cancer

**DOI:** 10.1111/jcmm.14042

**Published:** 2018-11-23

**Authors:** Shujun Zhang, Lutao Du, Lishui Wang, Xiumei Jiang, Yao Zhan, Juan Li, Keqiang Yan, Weili Duan, Yinghui Zhao, Lili Wang, Yunshan Wang, Yuliang Shi, Chuanxin Wang

**Affiliations:** ^1^ Department of Clinical Laboratory The Second Hospital of Shandong University Jinan Shandong China; ^2^ Tumor Marker Detection Engineering Laboratory of Shandong Province Jinan Shandong China; ^3^ Department of Clinical Laboratory Qilu Hospital of Shandong University Jinan Shandong China; ^4^ Department of Urology Qilu Hospital of Shandong University Jinan Shandong China; ^5^ School of Software Shandong University Jinan Shandong China

**Keywords:** biomarkers, bladder cancer, diagnosis, long non‐coding RNA, recurrence, serum exosomes

## Abstract

Exosomes are small membrane vesicles released by many cells. These vesicles can mediate cellular communications by transmitting active molecules including long non‐coding RNAs (lncRNAs). In this study, our aim was to identify a panel of lncRNAs in serum exosomes for the diagnosis and recurrence prediction of bladder cancer (BC). The expressions of 11 candidate lncRNAs in exosome were investigated in training set (n = 200) and an independent validation set (n = 320) via quantitative real‐time PCR. A three‐lncRNA panel (PCAT‐1, UBC1 and SNHG16) was finally identified by multivariate logistic regression model to provide high diagnostic accuracy for BC with an area under the receiver‐operating characteristic curve (AUC) of 0.857 and 0.826 in training set and validation set, respectively, which was significantly higher than that of urine cytology. The corresponding AUCs of this panel for patients with Ta, T1 and T2‐T4 were 0.760, 0.827 and 0.878, respectively. In addition, Kaplan‐Meier analysis showed that non‐muscle‐invasive BC (NMIBC) patients with high UBC1 expression had significantly lower recurrence‐free survival (*P* = 0.01). Multivariate Cox analysis demonstrated that UBC1 was independently associated with tumour recurrence of NMIBC (*P* = 0.018). Our study suggested that lncRNAs in serum exosomes may serve as considerable diagnostic and prognostic biomarkers of BC.

## 1 INTRODUCTION

Bladder cancer (BC) is the most common carcinoma of the urinary tract, and there are approximately 80 500 new cases and 32 900 BC‐related deaths in China in 2015.^1^, ^2^ Around 70% of BC patients are diagnosed as a non‐muscle‐invasive BC (NMIBC) characterized by a high recurrence rate, while the remaining 30% were diagnosed as a muscle‐invasive BC (MIBC) with poor prognosis.^3^ The standard of BC diagnosis includes cystoscopy and pathologic examination of biopsy specimens, both of which are invasive and relatively expensive.^4^ Urine cytology has good sensitivity for detection of high‐grade bladder tumours, but it unfits for low‐grade disease, and the recognition accuracy is highly dependent on the expertise of the pathologist.^5^ Additionally, current biomarkers, such as nuclear matrix protein 22 (NMP22), bladder tumour antigen and cytokeratin, have limited utility in the early examination of BC due to the lack of diagnostic sensitivity and specificity.^6^ Therefore, it is imperative to develop new biomarkers with high sensitivity and specificity for the diagnosis of BC.

Long non‐coding RNAs (lncRNAs) are a class of transcripts longer than 200 nucleotides with limited protein coding potential.^7^, ^8^ Accumulating evidences show that lncRNAs are involved in tumour initiation and progression through regulating associated gene expressions at the transcriptional,^9^, ^10^ posttranscriptional,^11^, ^12^ or epigenetic levels.^13^, ^14^ The aberrant lncRNAs have been reported as potential markers for diagnosis and prognosis of cancers. For example, increased expression of ZEB1‐AS1 promotes tumour metastasis and predicts poor prognosis in hepatocellular carcinoma,^15^ while overexpression of CRNDE‐h has been proposed as a potential novel molecular marker for colorectal cancer.^16^ These studies indicate that lncRNAs can serve as minimally invasive biomarkers of diagnosis and prognosis in different tumours.

Exosomes (70‐120 nm) are endosome‐derived microvesicles that are secreted from many cell types and involved in intercellular communication by transmitting intracellular cargoes, such as proteins, lipids and nucleic acids, including lncRNA.^17^ Studies have suggested that exosomes secreted from tumour tissues or cells can be transferred to the circulation through various model systems.^18^ Certain lncRNAs within exosomes have been described as candidate biomarkers in some tumours. For instance, lncARSR (lncRNA activated in RCC with sunitinib resistance) is highly expressed in patients with renal cancer, and it is correlated with clinically poor response to sunitinib.^19^ Plasma lncRNA00152 is protected by exosomes as a potential stable biomarker for gastric cancer.^20^ These findings suggest the potential application of these exosomal lncRNAs as biomarkers for the detection of malignant tumours. However, circulating exosomal lncRNAs have not been well evaluated as biomarkers for diagnosis or monitoring of BC.

Based on above‐mentioned findings, we evaluated the serum exosomal expressions of 11 lncRNAs (PCAT‐1, SPRY4‐IT1, MALAT1, UCA1, TUG1, UBC1, GHET1, H19, SNHG16, MEG3 and BC039493) which have been previously reported to be differently expressed in BC tissues. Data showed that three potential lncRNA markers were significantly up‐regulated in BC serum exosomes. Next, a panel consisting of these three lncRNAs was constructed to assess its diagnostic performance in BC, and the correlations between the expressions of three lncRNAs and clinicopathological features and prognosis of BC were further verified.

## 2 MATERIALS AND METHODS

### 2.1 Collection of serum samples

All serum samples were collected from Qilu Hospital of Shandong University, and informed consent was obtained from every participant. The sampling procedure was approved by the Clinical Research Ethics Committee of Qilu Hospital affiliated to Shandong University. The BC individuals did not receive any preoperative therapies before sample collection. BC patients were diagnosed by biopsy or histopathology, while tumour was staged and graded according to the WHO2004 grading scheme and the 2002UICC TNM classification. All clinicopathological data for the BC samples, including age, sex, clinical stage and histological grade, were obtained from the clinical and pathological records. Control participants without history of BC were recruited from a large pool of individuals seeking a routine health checkup at the Healthy Physical Examination Centre of Qilu Hospital, Shandong University. Table S1 summarizes the pathological and clinical data for the participants.

A total of 520 serum samples were collected, including 260 healthy serum samples and 260 BC serum samples. Serum was collected within 2 hours from coagulation‐promoting vacuum tubes and sequentially centrifuged at 1200 *g* for 5 minutes and then 9600 *g* for 5 minutes at 4°C to remove the cell debris. Subsequently, the supernatants were aliquoted into RNase‐free Eppendorf tubes and stored at −80°C prior to further analysis.

Non‐muscle‐invasive BC patients were followed up every 3 months during the first 2 years and then every 6 months thereafter. The date of the latest record retrieved was 20 June 2016. The median follow‐up time was 62.5 months (range: 5‐76 months). In addition, 10 BC patients were excluded due to incomplete follow‐up information.

### 2.2 Urine cytology

Urine samples were collected before cystoscopic examination and any other treatments, and centrifuged at 1300 *g* for 10 minutes. The sediments were used for cytological analysis, and the diagnosis was confirmed by two cytopathologists.

### 2.3 Exosome isolation

After serum sample was collected as referred above, 63 μL ExoQuick^™^ solution (EXOQ5A‐1; SBI System Biosciences, USA) was mixed well with 250 μL supernatant, followed by 30 minutes of incubation at 4°C. Subsequently, the mixture was centrifuged twice at 4°C (1500 *g* for 30 minutes and 1500 *g* for 5 minutes), and supernatants were discarded. The exosome pellets were resuspended in 50 μL PBS and stored at −80°C prior to further analysis.

### 2.4 Transmission electron microscopy

Isolated exosomes were first resuspended in PBS, and then a 15 μL aliquot was absorbed onto carbon‐coated Cu grids for 1 minute. Subsequently, the grids were dyed using 15 μL of 2.0% uranyl acetate for 1 minute and allowed to dry for 15 minutes. The morphology of isolated exosomes was identified by transmission electron microscopy (TEM; G2 spititi FEI; Tecnai).

### 2.5 Nanoparticle tracking analysis

Absolute size distribution and concentration of exosomes were determined using nanoparticle tracking analysis (NTA). Exosomes were diluted with PBS (1:1000) and mixed well, then the diluted exosomes were injected into the ZETASIZER Nano series‐Nano‐ZS instrument (Malvern, UK), and particles were automatically tracked and sized based on Brownian motion and the diffusion coefficient. NTA was performed under conditions of 25 frames/s and measurement time of 60 seconds. The detection threshold was similar in all the samples.

### 2.6 Western blotting analysis

Total exosome protein was extracted with RIPA extraction reagent (Thermo Fisher, USA) supplemented with a protease inhibitor cocktail (Roche, USA) at a ratio of 100:1. Protein concentration was determined by BCA protein assay kit (Thermo Fisher, USA). Equal amounts of proteins (approximately 30 μg) were subjected to 10% SDS‐PAGE and then transferred onto a polyvinylidene fluoride (PVDF) membrane (GE Healthcare, Piscataway, NJ, USA). The membrane was blocked with 5% non‐fat milk in TBST buffer and incubated with the primary antibodies against CD9 (1:1000, 13174S; CST) and TSG101 (1:1000, Ab83; Abcam) overnight at 4°C. Subsequently, the blot was washed with TBST, followed by incubation with HRP‐conjugated goat antimouse or goat anti‐rabbit secondary antibody (1:5000; Santa Cruz Biotechnology) at room temperature for 1 hour. The immunoreactive bands were visualized with Immobilon^™^ Western Chemiluminescent HRP Substrate (Millipore).

### 2.7 RNA extraction and reverse transcription

Total RNA was extracted from exosome and exosome‐depleted supernatant (EDS) using miRNeasyMicro Kit (Qiagen). Concentration and integrity of total RNA were evaluated using NanoDrop spectrophotometer (Thermo Fisher Scientific, Waltham, MA, USA). Purified RNA was reversely transcribed into cDNA using the PrimeScript^™^ RT reagent kit (Takara, Dalian, Liaoning, China) in a 20‐μL reaction system consisting of 200 ng template, 4 μL of 5× PrimeScript Buffer, 1 μL of PrimeScript RT Enzyme MixI, 1 μL of OligodT Primer and RNase‐free ddH_2_O. The mixture was centrifuged briefly and incubated at 37°C for 30 minutes, followed by 85°C for 5 seconds and 4°C for 60 minutes.

### 2.8 Quantitative real‐time polymerase chain reaction

Quantitative real‐time (qRT)‐PCR was performed in a 25‐μL reaction system containing 2 μL of diluted cDNA, 12.5 μL of SYBR^®^ Premix ExTaq^™^ (Takara), 0.5 μL of ROX Reference Dye α, 0.75 μL of gene‐specific forward and reverse primers (10 μmol/L) and 8.5 μL of RNase‐free ddH_2_O on a CFX‐96 real‐time PCR System according to the manufacturer's instructions. Table S2 lists the primer sequences used in this study. Briefly, after an initial denaturation step at 95°C for 30 seconds, amplifications were carried out with 42 cycles at a melting temperature of 95°C for 5 seconds and an annealing temperature of 60°C for 30 seconds. All experiments were conducted in triplicate, and no‐template controls were included in each run. The specificity of amplicons was confirmed by melting curve analysis. GAPDH was used as a reference gene. The relative expression levels of target genes were calculated using the 2^−∆∆CT^ method.

### 2.9 Statistical analysis

All statistical analyses were performed with SPSS 17.0 (IBM, SPSS, Chicago, IL, USA) and GraphPad Prism 5.0 (GraphPad Software, LaJolla, CA, USA). The distribution of each group was determined by Kolmogorov‐Smirnov test. Nonparametric Mann‐Whitney *U* tests were employed to compare the expression levels of lncRNAs between BC patients and healthy controls. ROC and AUC were used to evaluate the diagnostic performance of the selected lncRNAs by MedCalc 15.2.2 (MedCalc, Mariakerke, Belgium). MATLAB software (MATLAB, R2013a) was used for logistic regression analysis to establish lncRNA panel. Survival curves were plotted according to the Kaplan‐Meier method, with the log‐rank test applied for comparison. The independent prognostic factors were evaluated by the Cox proportional‐hazards regression model. All tests were two‐sided, and a *P*‐value of <0.05 was considered as statistically significant.

## 3 RESULTS

### 3.1 Characterisation of purified serum exosomes

**Figure 1:**
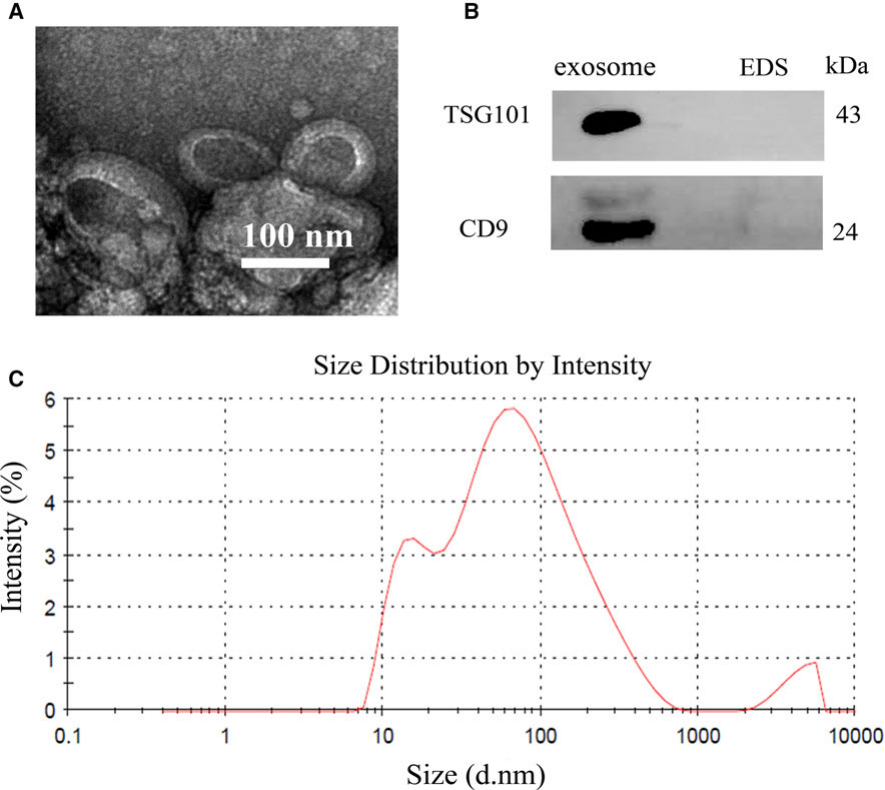
Identification of serum exosomes. (A) Representative TEM images of serum exosomes as indicated by the arrows. Scale bar, 100 nm. (B) Western blotting analysis of TSG101 and CD9 in exosomes and EDS. (C) NTA of the size distribution and number of exosomes

Exosomes are characterized by their conserved size and density as well as the presence of specific protein markers. To ensure that isolated exosomes from serum were recovered and intact, TEM was used to confirm the morphology of exosomes, which should be revealed as spherical vesicles with double layer membrane structure and diameters about 100 nm (Figure 1A). Western blotting analysis was used to examine the expressions of exosomal markers at the protein level. CD9 and TSG101 could be detected in the exosome samples but not in the EDS (Figure 1B). NTA showed the size distribution of exosomes (Figure 1C). Taken together, these results suggested that exosomes were successfully isolated from serum.

### 3.2 LncRNA marker identification from serum exosomes using a three‐step study

**Figure 2:**
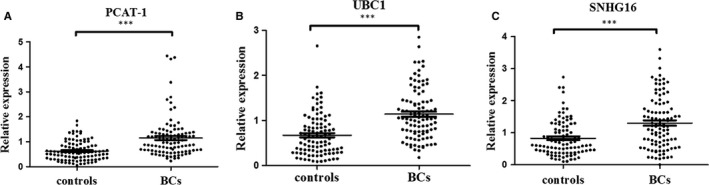
Relative expressions of three selected lncRNAs in serum exosome. Expression levels of serum exosomal PCAT‐1 (A), UBC1 (B) and SNHG16 (C) in BC patients (n = 100) and healthy controls (n = 100) using qRT‐PCR assay in the training set. Data are presented as 2^−∆∆Ct^. ***P < 0.001

To define BC‐associated lncRNA markers, we devised and carried out a three‐step case‐control strategy. In step I, we selected 11 lncRNAs (PCAT‐1, SPRY4‐IT1, MALAT1, UCA1, TUG1, UBC1, GHET1, H19, SNHG16, MEG3 and BC039493) as candidate targets, which have been previously reported to be differently expressed in BC tissues.^21^, ^22^, ^23^, ^24^, ^25^, ^26^, ^27^, ^28^, ^29^, ^30^ In step II, we quantified the expressions of 11 candidate lncRNAs from serum exosomes in 50 BC patients and 50 controls using the qRT‐PCR. The result revealed that three (PCAT‐1, UBC1 and SNHG16) of the 11 lncRNAs had a statistically increased expression in BC patients compared with the healthy donors (Table S3). To further validate these three lncRNAs identified in step II, we carried out the step III analysis by qRT‐PCR in an additional 50 BC cases and 50 healthy controls. Consequently, we found that the expressions of these three lncRNAs were also significantly higher in BC serum exosomes compared with the healthy controls (Figure 2A‐C, Table 1). We defined these 200 samples tested in step II and step III as the training set.

**Table 1:** Relative expression of three serum exosomal lncRNAs in BCs and controls in training set and validation set [median (interquartile range)]

lncRNA	Training set			Validation set		
	Controls (n = 100)	BCs (n = 100)	*P*‐value	Controls (n = 160)	BCs (n = 160)	*P*‐value
PCAT‐1	0.55 (0.31‐0.85)	0.97 (0.63‐1.35)	<0.001	0.60 (0.35‐1.00)	1.01 (0.72‐1.45)	<0.001
UBC1	0.59 (0.31‐0.92)	1.05 (0.74‐1.43)	<0.001	0.46 (0.24‐0.78)	0.86 (0.51‐1.32)	<0.001
SNHG16	0.66 (0.41‐1.20)	1.20 (0.66‐1.85)	<0.001	0.45 (0.28‐0.80)	0.92 (0.61‐1.35)	<0.001

### 3.3 Characterisation of identified three serum exosomal lncRNAs

**Figure 3:**
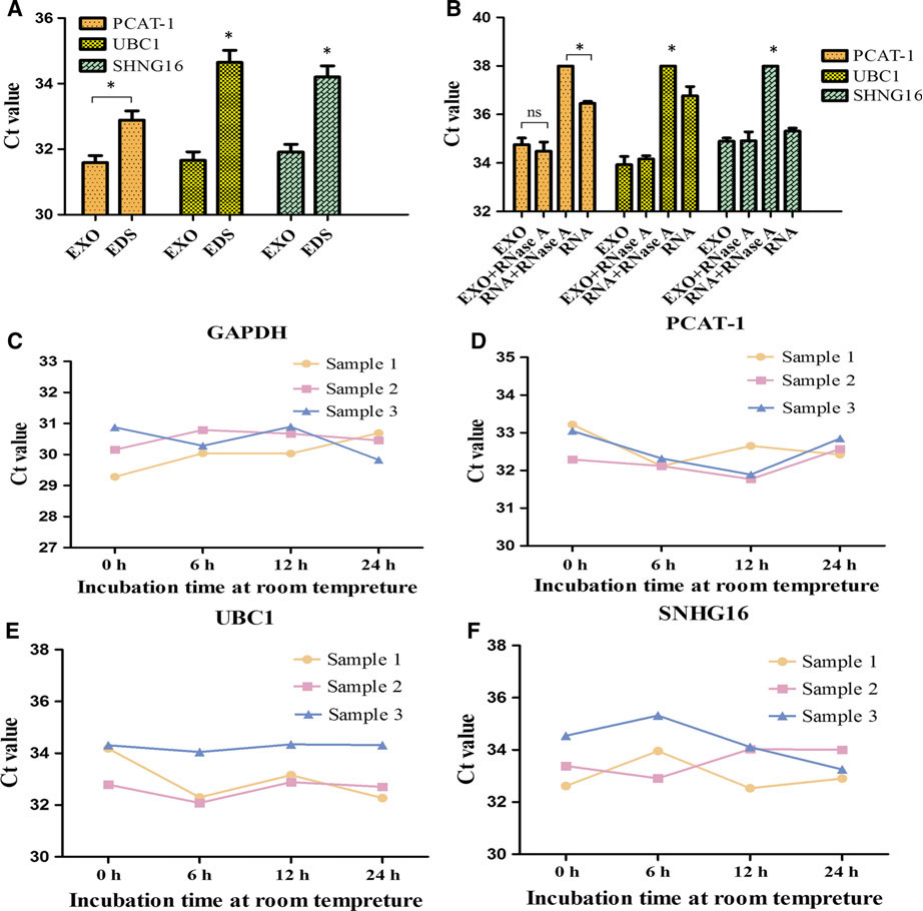
General characterisation of the three exosomal lncRNAs. (A) Expression levels of PCAT‐1, UBC1 and SNHG16 from serum exosome (EXO) and EDS. (B) qRT‐PCR analysis of the three lncRNAs in the exosomes or isolated nucleic acids treated with RNase A (2 mg/mL). (C‐F) The expressions of the three serum exosomal lncRNAs and GAPDH when incubated at room temperature. *P < 0.05, NS, not significant

To confirm whether serum lncRNAs were exclusively distributed into exosomes just like microRNAs,^31^ we compared the expression levels of three lncRNAs between exosome and EDS. The result showed that the expressions of PCAT‐1, UBC1 and SNHG16 in exosomes were higher than those in EDS (Figure 3A). Our finding demonstrated that lncRNAs in serum were distributed mainly in the exosomes.

Next, we investigated the stability of exosomal lncRNAs. The expression levels of lncRNAs in exosomes remained unchanged upon RNase A treatment (Figure 3B). In room‐temperature incubation test, the exosome aliquots were maintained at room temperature for 0, 6, 12 and 24 hours. No significant changes were found for the expressions of three lncRNAs and GAPDH at different time‐points (Figure 3C‐F). Taken together, these results indicated that the three lncRNAs mainly existed in exosomes, and exosomes were the main factor for protecting serum lncRNAs.

### 3.4 A trial detection of BC using a three‐lncRNA panel

**Figure 4:**
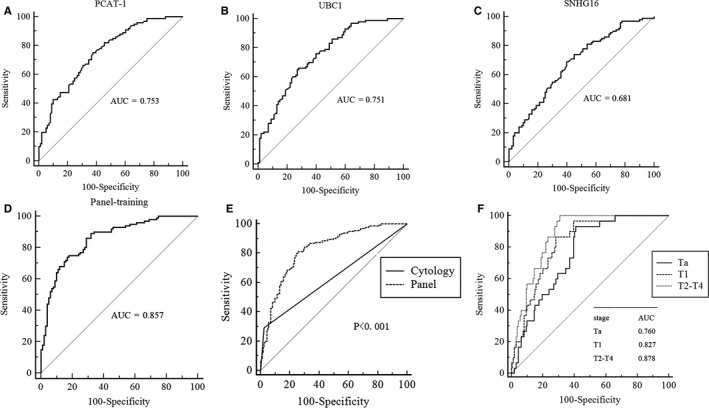
Diagnostic performance of three‐lncRNA panel for BC. (A‐C) ROC curve analysis using PCAT‐1, UBC1 and SNHG16 for BC detection in the training set. (D) ROC curves of the three‐lncRNA diagnostic panel in the training set. (E) Comparison of diagnostic performance between three‐lncRNA panel and urine cytology for BC detection in additional validation set. (F) ROC curves showing the diagnostic performance of the three‐lncRNA panel for Ta, T1 and T2‐T4 in the validation set

To exploit the potential ability of BC classification based on the three lncRNAs, ROC curves and AUC were calculated for each molecular. In the training cohort, BCs could efficiently be distinguished from controls using PCAT‐1, UBC1 and SNHG16 (PCAT‐1: AUC = 0.753; UBC1: AUC = 0.751; SNHG16: AUC = 0.681) (Figure 4A‐C). Next, multivariate logistic regression model was made by logistic regression to evaluate whether a combination of markers could optimize the separation of the group with cancer vs the group without cancer. In the light of the AUC, the three lncRNAs with an individual ability to separate the groups were included. The combined AUC was 0.857 (95% CI: 0.801‐0.903) (Figure 4D), and the accuracy of classifying patients into the correct groups was 81.5% (sensitivity 0.85, specificity 0.78), indicating that the three‐lncRNA panel could be used to accurately differentiate BC individuals.

We further analysed the expression levels of the three lncRNAs using an independent validation cohort, including 160 BC patients and 160 healthy controls. In this assessment analysis, the expressions of three lncRNAs in the validation set were consistent with those in the training set (Table 1, Figure S1A‐C). The AUC of the lncRNA panel was 0.826 (95% CI: 0.780‐0.866, sensitivity = 80.00%, specificity = 75%, Figure 4E). Using samples in the validation set, we compared the diagnostic efficacy of the three‐lncRNA panel with urine cytology. The AUC values of our three‐lncRNA panel were markedly higher than those of urine cytology (0.574, 95% CI: 0.432‐0.708) when discriminating BC patients from healthy controls (Figure 4E). Additionally, ROC curves also showed that this panel was sensitive and specific to distinguish Ta, T1 and T2‐T4 from healthy controls (Figure 4F). Overall, our results indicated that the three‐lncRNA panel was able to differentiate BC cases from healthy controls with an excellent accuracy, suggesting its potential to be a better biomarker for BC diagnosis than urine cytology.

### 3.5 Diagnostic performance of the three lncRNAs in distinguishing MIBC from NMIBC

**Figure 5:**
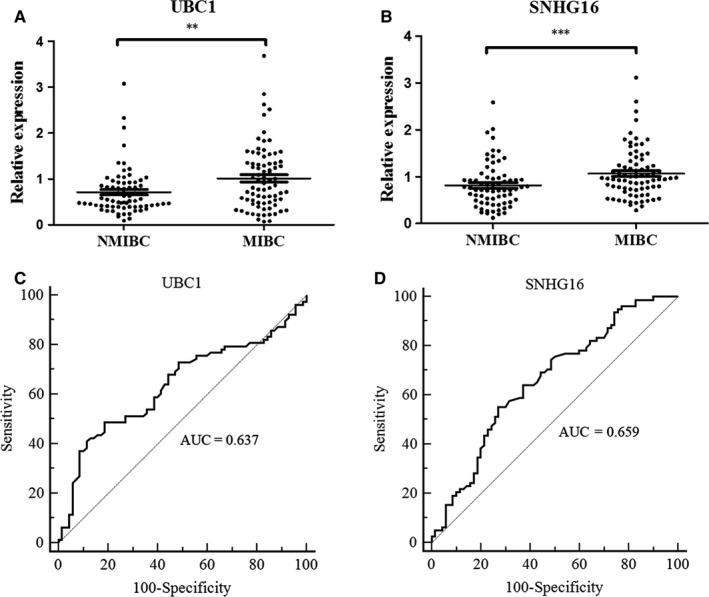
Diagnostic performance of three lncRNAs for distinguishing MIBC from NMIBC. (A, B) Expression levels of serum exosomal UBC1 and SNHG16 in patients with NMIBC or MIBC using RT‐qPCR assay in the validation set. (C, D) ROC curve analysis using UBC1 and SNHG16 for MIBCs vs NMIBCs. **P < 0.01, ***P < 0.001

Methods to identify NMIBC and MIBC patients have previously relied on conventional histopathologic evaluation. These histopathologic features have failed to properly risk stratify these patients.^32^ Therefore, to determine whether the expression levels of three lncRNA were related to BC progression, we analysed the association between these three lncRNAs and clinicopathological status in BC patients of validation set. Table 2 and Figure 5A,B shows that UBC1 and SNHG16 were significantly up‐regulated in MIBC and correlated with tumourous invasion (*P* = 0.009 and *P* = 0.0009, respectively). The corresponding AUCs of such two lncRNAs were 0.637 and 0.659, respectively (Figure 5C,D). In addition, statistical analysis also represented a moderate correlation between UBC1 expression and lymph node metastasis (*P* = 0.005), and higher PCAT‐1 level was correlated with higher tumour grade (*P* = 0.01) (Table 2). However, no significant associations were found between the three exosomal lncRNAs and age or sex.

**Table 2:** Correlations between exosomal lncRNA concentrations and clinicopathological characteristics of patients with BC in validation set [median (interquartile range)]

Parameters	Total cases	PCAT‐1	*P‐*value	UBC1	*P*	SNHG16	*P‐*value
Age							
<60	51	1.27 (0.96‐1.67)	0.40	0.81 (0.45‐1.40)	0.73	0.92 (0.59‐1.45)	0.42
≥60	109	1.00 (0.72‐1.40)		0.81 (0.47‐1.64)		0.92 (0.49‐1.31)	
Sex							
Male	125	1.02 (0.72‐1.68)	0.09	0.92 (0.51‐1.33)	0.23	0.90 (0.49‐1.26)	0.29
Female	35	1.00 (0.69‐1.24)		0.77 (0.49‐1.02)		0.78 (0.45‐1.04)	
Tumour stage							
NMIBC(Ta‐T1)	84	0.91 (0.48‐1.30)	0.23	0.52 (0.39‐0.92)	0.009	0.72 (0.45‐0.98)	0.0009
MIBC(T2‐T4)	76	0.72 (0.52‐1.01)		0.86 (0.47‐1.34)		0.96 (0.73‐1.28)	
Tumour grade							
Low grade	66	0.80 (0.42‐1.22)	0.01	0.89 (0.58‐1.41)	0.30	0.93 (0.64‐1.46)	0.51
High grade	94	0.96 (0.63‐1.64)		0.82 (0.46‐1.30)		0.92 (0.60‐1.23)	
Lymph node metastasis							
No	152	0.92 (0.65‐1.64)	0.61	0.87 (0.51‐1.33)	0.005	0.94 (0.61‐1.36)	0.33
Yes	8	1.01 (0.60‐1.25)		1.53 (1.09‐2.50)		0.77 (0.57‐0.98)	

### 3.6 Correlation between the expression levels of three lncRNAs and prognosis of NMIBC patients

**Figure 6:**
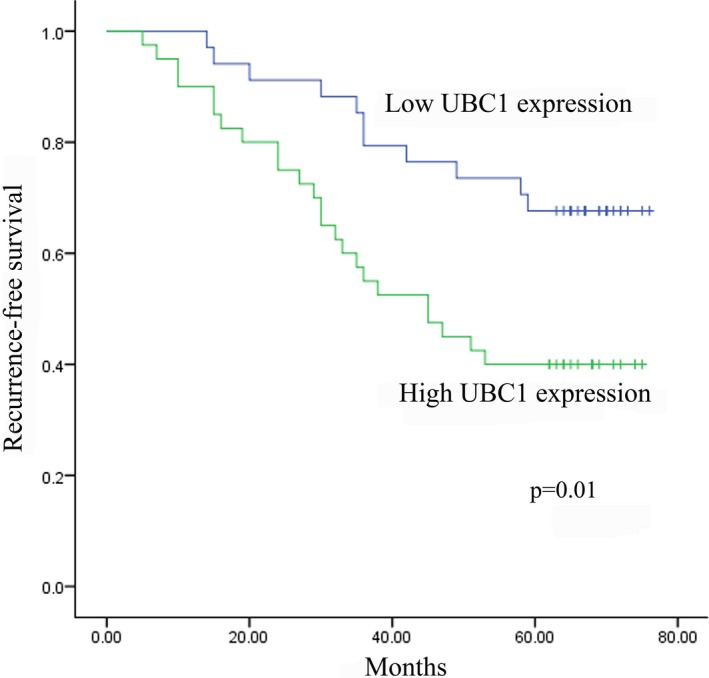
UBC1 expression is correlated with RFS in NMIBC patients. Kaplan‐Meier curve showed that high UBC1 (A) expression was associated with a worse RFS in NMIBC patients in the validation set

Non‐muscle‐invasive BC is characterized by the frequent recurrence; therefore, cystoscopic surveillance is essential after tumour resection, intravesical prophylaxis or treatment and during the maintenance prophylaxis period.^33^ However, cystoscopy is invasive and relatively expensive. Thus, we aimed to further examine whether the expression levels of these three lncRNAs were correlated with the recurrence of NMIBC as effective non‐invasive biomarkers, which might be useful adjunct to conventional cystoscopy. The median follow‐up time for recurrence‐free survival (RFS) was 62.5 (range 5‐76) months. The Kaplan‐Meier analysis revealed that high UBC1 expression in serum exosomes was significantly correlated with a reduced RFS compared with those with low UBC1 expression in NMIBC patients (n = 74) (*P* = 0.01; Figure 6A). However, the expression levels of PCAT‐1 and SNHG16 had no correlation with RFS (Figure S2A,B). Moreover, univariate and multivariate Cox regression analyses showed that UBC1 expression (*P* = 0.018) and tumour stage (*P* = 0.035) were independent prognostic factors for RFS of NMIBC (Table 3). These data suggested that high UBC1 expression in serum exosome was correlated with RFS in NMIBC patients.

**Table 3:** Univariate and multivariate Cox proportional‐hazards regression model analysis of RFS in patients with NMIBC in validation set

Parameters	Categories	Univariate analysis		Multivariate analysis	
		HR (95% CI)	*P*‐value	HR (95% CI)	*P*‐value
Age	<65 vs ≥65	1.355 (0.693‐2.650)	0.375		
Sex	Male vs female	0.909 (0.353‐2.345)	0.844		
Tumour grade	Low vs high	0.752 (0.328‐1.722)	0.500		
Tumour stage	Ta vs T1	2.146 (1.101‐4.183)	0.025	2.054 (1.052‐4.010)	0.035
PCAT‐1 expression	Low vs high	1.425 (0.729‐2.784)	0.300		
UBC1 expression	Low vs high	2.460 (1.202‐5.033)	0.014	2.371 (1.157‐4.857)	0.018
SNHG16 expression	Low vs high	1.721 (0.873‐3.390)	0.117		

## 4 DISCUSSION

In the present study, we identified three up‐regulated serum exosomal lncRNAs (PCAT‐1, UBC1 and SNHG16) in BC and further designed a three‐lncRNA panel as a novel diagnostic biomarker for BC based on a multivariate logistic regression model. Moreover, this panel was significantly superior to traditional urine cytology in terms of diagnostic accuracy. In addition, our data proved that these lncRNAs in serum were mainly stored in the exosomes. Among these three lncRNAs, UBC1 and SNHG16 could be used to distinguish MIBC from NMIBC. UBC1 was also identified as an independent prognostic factor for RFS in NMIBC. These results suggested that serum exosomal lncRNAs could be used as an easier and faster non‐invasive approach for diagnosis and recurrence prediction of BC.

Liquid biopsy has been reported to be more convenient and has higher sensitivity for cancer diagnosis compared with traditional imaging and biopsy strategies.^34^ As a promising alternative to liquid biopsy, tumour‐derived circulating exosomes have intrigued increasing interest in non‐invasive cancer diagnosis and monitoring of treatment response.^35^ Previous studies have shown that exosomes contain proteins, miRNAs and lncRNAs.^36^ LncRNAs can be protected by exosomes from degradation in the circulation, and they are useful for cancer diagnosis at the early stage.^37^ For instance, lncRNA‐p21 in exosomes may help distinguish prostate cancer from benign disease.^38^ Our previous study has revealed that serum exosomal lncRNA CRNDE‐h can be used as a novel biomarker for diagnosis and prognosis of colorectal cancer.^39^ However, little is known about possible application of circulating exosomal lncRNAs in BC. For this reason, we firstly confirmed the high purity and enrichment of exosomes in serum of BC patients by using TEM and Western blotting. Then we compared the expressions of serum exosomal lncRNAs between BC patients and healthy controls for the first time and identified three significantly up‐regulated lncRNAs (PCAT‐1, UBC1 and SNHG16) from 11 candidate lncRNAs. We established a diagnostic panel based on these three lncRNAs by logistic regression, which was able to differentiate BC cases from healthy controls with an excellent accuracy and might be a better biomarker than urine cytology. In addition, we clarified that these three lncRNAs were mainly stored in serum exosomes and had significant higher levels than those in the EDS. Moreover, we evaluated the stability of the three exosomal lncRNAs, and the results indicated that these lncRNAs were still stable when incubated for long time at room temperature even direct RNase A digestion of exosomes. These findings suggested that the membranaceous structures of exosomes could indeed protect these molecules from physical degradation. In view of the stability of three lncRNAs as well as the simplicity and reproducibility of obtaining serum sample, exosomal lncRNAs from serum could be used as biomarkers in clinical practice.

Long non‐coding RNAs are emerging as important regulatory molecules in tumour‐suppressor and oncogenic pathways. These three lncRNAs have been previously reported as the aberrantly expressed lncRNAs in BC and other cancer tissue samples. Liu et al^22^ have demonstrated that PCAT‐1 is up‐regulated in BC tissue, and silencing PCAT‐1 inhibits BC cell growth and induces apoptosis. Prensner et al^40^ have determined that PCAT‐1 regulates cmyc post‐transcriptionally by interfering with the regulation of MYC by miR‐34a to promote prostate cell proliferation. LncRNA UBC1 is physically associated with polycomb repressive complex 2, and it acts as a negative prognostic factor for lymph node metastasis and survival of BC patients, playing important roles in BC cell proliferation, migration, invasion, colony formation, tumourigenicity and metastatic potential.^21^ Previous studies have demonstrated that SNHG16 is up‐regulated and associated with poor patient outcome in neuroblastoma and invasiveness of BC.^28^, ^41^ Studies have also found that SNHG16 is overexpressed in early CRC, and knockdown of SNHG16 induces apoptotic death and increases cellular migration.^42^ These study results supported that the biomarkers we have identified are involved in BC tumourigenesis and progression and further reinforced the use of lncRNAs as potential diagnostic indicators.

In this study, we also investigated the association between these three lncRNAs and the recurrence of NMIBC. The Kaplan‐Meier analysis revealed that high UBC1 expression was associated with a higher recurrence rate in NMIBC. Furthermore, univariate and multivariate Cox model analyses confirmed that UBC1 was an independent risk factor for RFS in NMIBC. Collectively, we, for the first time, indicated that exosomal UBC1 was a useful prognostic biomarker to help identify patients with a higher risk of NMIBC recurrence.

Although we constructed a promising three‐lncRNA panel for BC diagnosis, there were some limitations in our study. The study was performed at a single centre in Qilu Hospital with relatively limited sample size. Moreover, functional analysis was also required to elucidate the biological mechanisms in BC and to confirm the possible role of exosomal lncRNAs in BC identified by both bioinformatic analysis and literature review.

Taken together, we established a distinctive serum exosomal lncRNA signature that might represent a new complementary marker for BC diagnosis. Moreover, we identified that UBC1 expression was a useful prognostic marker for RFS in BC. Further studies, including larger clinical samples, multicentre study and functional analysis, are required to support the importance of these lncRNAs as noninvasive markers in BC.

## CONFLICT OF INTEREST

No potential conflicts of interest were disclosed.
