# Supplementary figures and images for: Evaluation of serum exosomal LncRNA‐based biomarker panel for diagnosis and recurrence prediction of bladder cancer
Source: J Cell Mol Med. 2018 Nov 23;23(2):1396–405. doi: 10.1111/jcmm.14042 (PMC6349164; doi:10.1111/jcmm.14042)

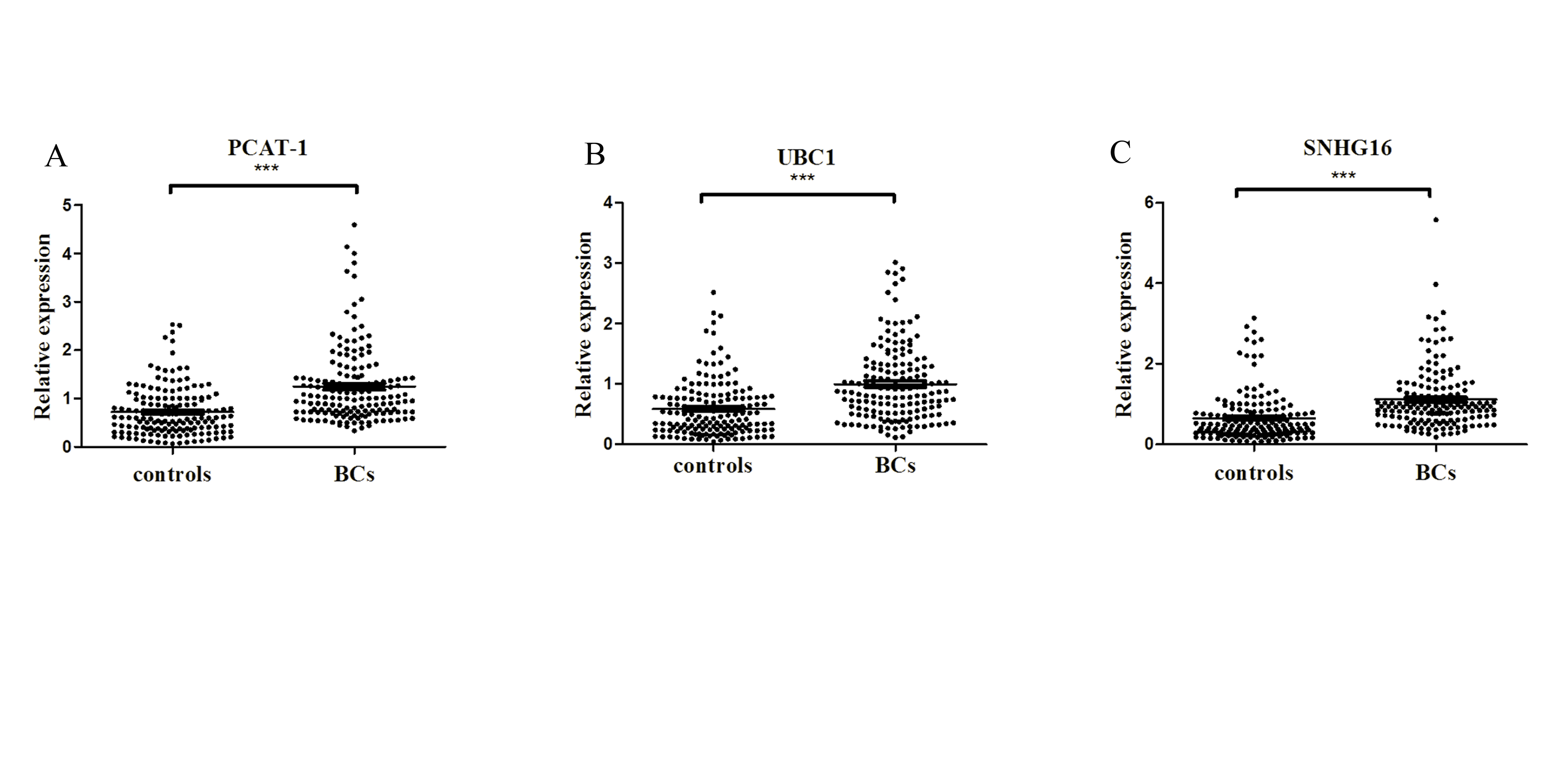

Supplement: Supplementary file 1 [file JCMM-23-1396-s001.tif]

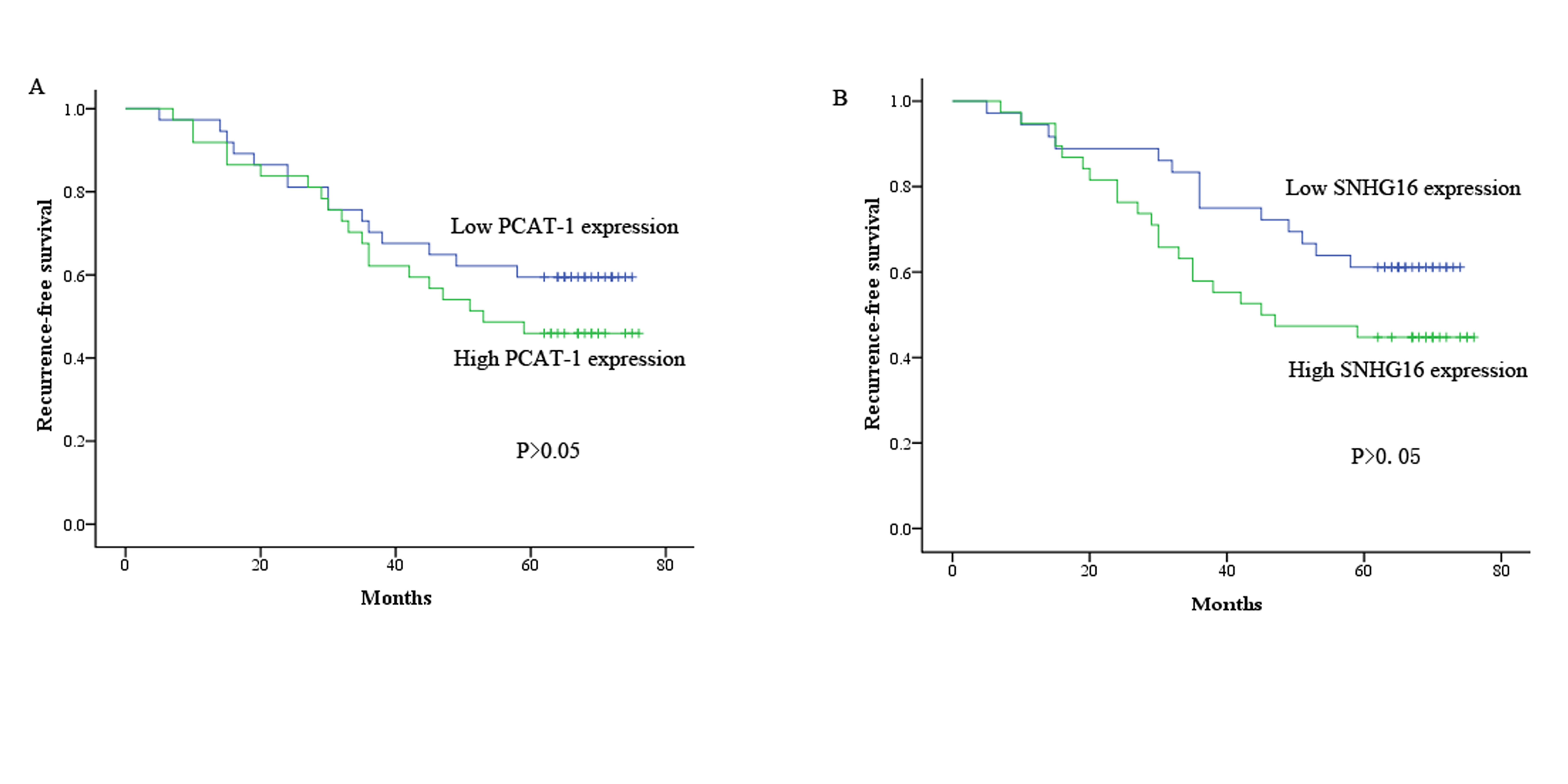

Supplement: Supplementary file 2 [file JCMM-23-1396-s002.tif]
