# Supplementary material for: Evaluation of serum exosomal LncRNA‐based biomarker panel for diagnosis and recurrence prediction of bladder cancer
Source: J Cell Mol Med. 2018 Nov 23;23(2):1396–405. doi: 10.1111/jcmm.14042 (PMC6349164; doi:10.1111/jcmm.14042)
Supplement: Supplementary file 3 [file JCMM-23-1396-s003.docx]

**Table S1: Characteristics of study participants in training set and validation set**

| **Variale** | **Training set** | **Validation set** | ***p-*Value** |
| --- | --- | --- | --- |
|  | **（n=200）** | **（n=320）** |  |
| **Control (number)** | **100** | **160** |  |
| **Gender** |  |  | 0.341 |
| Male | 72 | 106 |  |
| Female | 28 | 54 |  |
| **Age** |  |  | 0.518 |
| <60 | 57 | 98 |  |
| ≥60 | 43 | 62 |  |
| **Tumor (number)** | **100** | **160** |  |
| **Gender** |  |  | 0.639 |
| Male | 81 | 125 |  |
| Female | 19 | 35 |  |
| **Age** |  |  | 0.421 |
| <60 | 37 | 51 |  |
| ≥60 | 63 | 109 |  |
| **Tumor stage** |  |  | 0.526 |
| Ta-T1 | 56 | 84 |  |
| T2-T4 | 44 | 76 |  |
| **Tumor grade** |  |  | 0.306 |
| \| Low grade \| \| --- \|   High grade | 48  52 | 66  94 |  |
| **Lymph Node Metastasis** |  |  | 0.137 |
| No | 90 | 152 |  |
| Yes | 10 | 8 |  |
