# Supplementary material for: Evaluation of serum exosomal LncRNA‐based biomarker panel for diagnosis and recurrence prediction of bladder cancer
Source: J Cell Mol Med. 2018 Nov 23;23(2):1396–405. doi: 10.1111/jcmm.14042 (PMC6349164; doi:10.1111/jcmm.14042)
Supplement: Supplementary file 4 [file JCMM-23-1396-s004.docx]

**Table S2:** Sequences of primers used for qRT-PCR in this study

| **Primers** | **Forward** | **Reverse** |
| --- | --- | --- |
| GAPDH | 5'-ACCCACTCCTCCACCTTTGAC-3' | 5'TGTTGCTGTAGCCAAATTCGTT-3' |
| PCAT-1 | 5'-GAGAGCTGACATAGGCACCC-3' | 5'-TCTCCACTGGTGTTCATGGC-3' |
| SPRY-IT1 | 5'-CCCCAGAGAGCCAAGTCATC-3' | 5'-CACAGGCTCCCATAACCCTC-3' |
| MALAT1 | 5' -AAAGCAAGGTCTCCCCACAAG-3' | 5' -GGTCTGTGCTAGATCAAAAGGCA-3' |
| UCA1 | 5'-CTCTCCATTGGGTTCACCATTC-3′ | 5'-GCGGCAGGTCTTAAGAGATGAG-3' |
| TUG1 | 5' -TAGCAGTTCCCCAATCCTTG-3' | 5' -CACAAATTCCCATCATTCCC-3' |
| UBC1 | 5'-CCTGCTTGGAAACTAATGACC-3' | 5'-AGGCTCAACTTCCCAGACTCA-3' |
| GHET1 | 5'-CCCCACAAATGAAGACACT-3' | 5'-TTCCCAACACCCTATAAGAT-3' |
| H19 | 5'-GCACCTTGGACATCTGGAGT-3' | 5'-TTCTTTCCAGCCCTAGCTCA-3' |
| SNHG16 | 5'-AATCGCCATGCGTTCTTTGG-3' | 5'-CAATCCTTGCAGTCCCATCG-3' |
| MEG3 | 5'-GGGCATTAAGCCCTGACCTT-3' | 5'-CCTTGGGGAGGGAAACACTC-3' |
| BC039493 | 5'-AATGCACTGCCTAGATGGAC-3' | 5'-CACCTGTTTTCTGGCTCTTCC-3' |
