# Supplementary material for: Evaluation of serum exosomal LncRNA‐based biomarker panel for diagnosis and recurrence prediction of bladder cancer
Source: J Cell Mol Med. 2018 Nov 23;23(2):1396–405. doi: 10.1111/jcmm.14042 (PMC6349164; doi:10.1111/jcmm.14042)
Supplement: Supplementary file 5 [file JCMM-23-1396-s005.docx]

Table S3: **Expression of 11 candidate serum exosomal lncRNAs in 50 BCs and 50 controls [median (interquartile range)]**

| lncRNA | controls | BCs | *p*-Value | lncRNA | Controls | BCs | *p*-Value |
| --- | --- | --- | --- | --- | --- | --- | --- |
| PCAT-1 | 0.59(0.30-1.0) | 1.12(0.66-1.51) | <0.001 | GHET1 | 1.11(0.59-2.17) | 0.98(0.60-1.83) | 0.6420 |
| SPRY-IT1 | 1.46(0.84-2.18) | 1.83(1.09-2.75) | 0.0817 | H19 | 0.52(0.38-1.02) | 0.84(0.43-1.66) | 0.0677 |
| MALAT1 | 0.58(0.36-1.01) | 0.79(0.49-1.07) | 0.0627 | SNHG16 | 0.74(0.51-1.29) | 1.42(0.80-1.78) | <0.001 |
| UCA1 | 0.48(0.28-0.78) | 0.41(0.30-0.98) | 0.6051 | MEG3 | 0.78(0.46-1.39) | 0.67(0.43-0.91) | 0.1723 |
| TUG1 | 2.02(1.12-3.21) | 2.47(1.44-4.11) | 0.0906 | BC039493 | 0.53(0.31-0.97) | 0.67(0.43-0.98) | 0.1926 |
| UBC1 | 0.66(0.35-0.96) | 1.02(0.71-1.65) | 0.001 |  |  |  |  |
